# Marine Carbonates in the Mantle Source of Oceanic Basalts: Pb Isotopic Constraints

**DOI:** 10.1038/s41598-018-33178-4

**Published:** 2018-10-08

**Authors:** P. R. Castillo, C. MacIsaac, S. Perry, J. Veizer

**Affiliations:** 1 Scripps Institution of Oceanography, UCSD, 9500 Gilman Drive, La Jolla, CA 92093 USA; 2 Department of Earth and Environmental Sciences, University of Ottawa, Ottawa, ON K1N 6N5 Canada

## Abstract

For almost fifty years, geochemists have been interpreting the clues from Pb isotopic ratios concerning mantle composition and evolution separately. The Pb isotopes of ocean island basalts (OIB) indicate that their mantle source is heterogeneous, most likely due to the presence of end-components derived from recycled crust and sediment. Some OIB have unusually high ^206^Pb/^204^Pb coming from one of the end-components with a long time-integrated high ^238^U/^204^Pb or μ (HIMU). Most OIB and many mid-ocean ridge basalts (MORB) also have high ^206^Pb/^204^Pb, indicating a HIMU-like source. Moreover, measured ^232^Th/^238^U (κ) for most MORB are lower than those deduced from their ^208^Pb/^204^Pb and ^206^Pb/^204^Pb. Such high μ and low κ features of oceanic basalts are inconsistent with the known geochemical behavior of U, Pb and Th and temporal evolution of the mantle; these have been respectively termed the 1^st^ and 2^nd^ Pb paradox. Here we show that subducted marine carbonates can be a source for HIMU and a solution to the Pb paradoxes. The results are consistent with the predictions of the marine carbonate recycling hypothesis that posits the Pb isotopes of oceanic basalts indicate a common origin and/or magma generation process.

## Introduction

**Figure 1:**
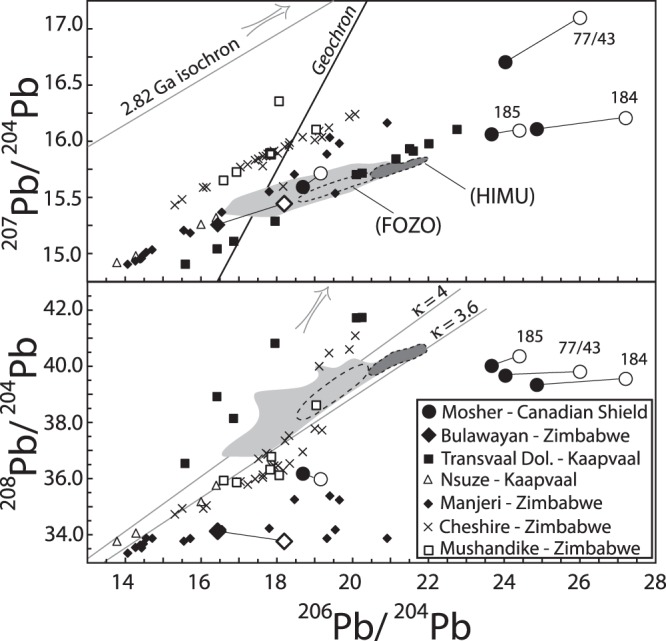
^206^Pb/^204^Pb versus A. ^207^Pb/^204^Pb and B. ^208^Pb/^204^Pb for Archaean limestones. Bulk stromatolitic limestones (solid symbols) are connected by tie lines to their respective carbonate fractions (open symbols). Analytical errors are smaller than the symbols used. Shown for reference are the carbonate fractions of other Archaean limestones (see text for sources of data), oceanic basalts (gray field), FOZO (dash area inside gray field), and HIMU end-component (dark gray field). The 2.82 Ga isochron in A. is the regression line for the extremely radiogenic (^206^Pb/^204^Pb = 30.2 to 104.4; ^207^Pb/^204^Pb = 19.4 to 34.1; ^208^Pb/^204^Pb = 42.514 to 74.402) Mushandike stromatolites^24^ (not shown).

Together with Sr isotopes, the Pb isotopes of OIB showed, for the first time, that the mantle is heterogeneous^1^, most likely due to the presence of enriched mantle 1 (EM1), enriched mantle 2 (EM2) and HIMU end-components from recycled crust and sediment^2,3^. High μ OIB from Saint Helena, Mangaia and Tubuai islands have unusually high ^206^Pb/^204^Pb (>20.0) and low ^87^Sr/^86^Sr (≤0.7028), and the bulk of OIB and many MORB also have high ^206^Pb/^204^Pb although higher ^87^Sr/^86^Sr (>0.7028). That is, almost all oceanic basalts have high ^206^Pb/^204^Pb that plot to the right of Geochron, a line in the ^206^Pb/^204^Pb versus ^207^Pb/^204^Pb diagram containing the current Pb isotopes of terrestrial materials, assuming the Earth remained a closed system (Fig. 1A). Both ^206^Pb and ^207^Pb increase due to the respective decay of radioactive ^238^U and ^235^U and, hence, the mantle source of oceanic basalts must have a long time-integrated (~b.y.) U/Pb > BSE refs^2–4^. Such a high U/Pb is unexpected because U is more incompatible than Pb and, thus, the ratio should be low at least in the depleted MORB mantle (DMM) source that has experienced repeated melt extraction. This has been termed the main or 1^st^ Pb paradox, and its proposed solutions include transfer of Pb into the core^4,5^, preferential retention of Pb relative to U in the continent^6,7^ or residual mantle sulfide^8^, hydrothermal transfer of Pb from mantle to continent^9,10^, U recycling into the mantle since Early Proterozoic^2,11^, and crustal contamination of an early-formed (ca. 4.5 Ga) mantle reservoir^12^. Notably, a majority of these solutions call for a decrease in Pb to increase U/Pb refs^2,3^. Moreover, the distinction between the sources of HIMU end-component, which has characteristically low ^87^Sr/^86^Sr, and HIMU-like characteristics of many oceanic basalts, which have variable and higher ^87^Sr/^86^Sr, has become blurred. A majority of the proposed solutions do not consider that the HIMU end-component is distinct because it must also have a long time-integrated, low Rb/Sr, as the decay of radioactive ^87^Rb increases ^87^Sr.

Measured κ (2.5 average) for most MORB are lower than those deduced from their ^208^Pb/^204^Pb and ^206^Pb/^204^Pb, which are fairly homogeneous and only slightly lower than bulk silicate Earth (BSE; κ ca. 4.0) refs^13,14^. The increase in ^208^Pb results from the decay of radioactive ^232^Th. The measured low κ of MORB indicates that the Th/U decrease from the original BSE κ in DMM may have occurred as recently as ca. 600 Ma ref.^13^. However, the Th-enriched continental crust derived from DMM is much older than this and, thus, the Th/U evolution of the mantle is a dilemma^2,13,14^. This has been termed the 2^nd^ Pb paradox, and its proposed solutions include a mantle BSE κ that decreased concurrently with continental extraction^15,16^, an upper mantle BSE κ that abruptly changed to 2.5 at 600 Ma^13^ and mantle recycling of U due to the Earth’s oxidizing condition since Early Proterozoic^2,11,14,17^.

Quite recently, we hypothesized that some Archaean marine carbonates provide the radiogenic ^206^Pb/^204^Pb, unradiogenic ^87^Sr/^86^Sr, low K_2_O, and high CaO of HIMU, as well as the overall positive correlation between CaO and ^206^Pb/^204^Pb in OIB ref.^18^. Our hypothesis is consistent with the Archaean-formed Δ^33^S anomaly in HIMU OIB ref.^19^ and the similar trace element contents of carbonatitic melt inclusions in diamonds and HIMU lavas^20^. We also proposed that the unusually radiogenic Pb isotopes of OIB (Fig. 1A) and 1^st^ and 2^nd^ Pb paradoxes are parts of a system of equations, and the higher concentration of U relative to Pb and Th in marine carbonates offers a self-consistent solution to these equations^21^. Such a holistic interpretation of Pb isotopes, however, assumes some Archaean marine carbonates formed in equilibrium with seawater containing U, but little to no Pb and Th, as well as high Sr but little to no Rb ref.^18^. This assumption is inconsistent with the anoxic Archaean ocean that should have prevented U mobilization in seawater and its eventual subduction into the mantle^2,11,14,17^.

To verify if some Archaean marine carbonates have high U/Pb and U/Th, we analyzed the Pb and Sr isotopes of the carbonate fractions of the few stromatolitic limestones that have the low ^87^Sr/^86^Sr of coeval seawater^22,23^. Notably, these carbonates preserve the composition of Archaean oceans, have long time-integrated low Rb/Sr and are generally unaltered, as alteration would have raised their ^87^Sr/^86^Sr refs^22,23^. Accordingly, the Pb isotopes of these unaltered samples should also be able to constrain their long time-integrated U, Th and Pb concentrations and ratios. We adopted this approach because the current U, Th, Pb, Rb, and Sr concentrations of many Archaean limestone outcrops have been modified by contamination and alteration^22–26^. We also analyzed their bulk Pb and Sr isotopes for comparison. Four samples (184, 185, 1977/7, and 1977/43) are from the 2.8 Ga Mosher Carbonate Formation in the Canadian Shield^27^ and one sample (187) is from the 3.0 to 2.6 Ga Bulawayan Supergroup in the Zimbabwe Craton^28^. The location and full description of the samples can be found in ref.^23^.

## Results

**Figure 2:**
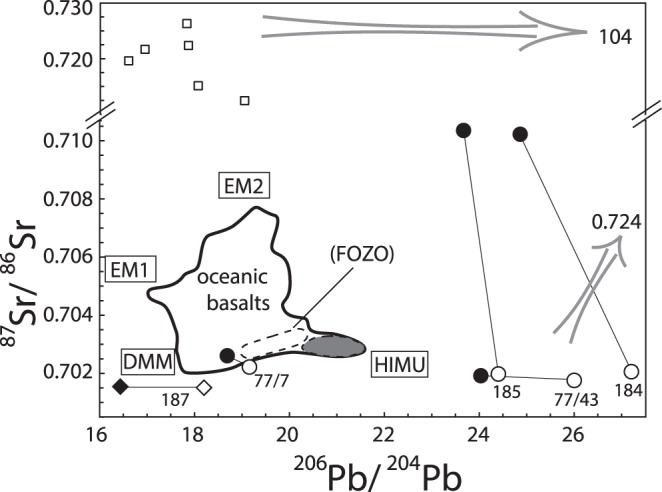
^206^Pb/^204^Pb versus ^87^Sr/^86^Sr for Archaean limestones. Symbols as in Fig. 1. Shown for reference are the carbonate fractions of the few Mushandike limestones that have ^87^Sr/^86^Sr data (gray arrows point to the approximate location of other samples), the field for oceanic basalts and locations of proposed mantle end-components.

The bulk limestone Pb isotopes are highly variable, but their carbonate fractions have systematically higher ^206^Pb/^204^Pb and ^207^Pb/^204^Pb (Table 1; Figs 1 and 2). On the other hand, the carbonate fractions of samples 184, 185 and 1977/7 have systematically lower ^87^Sr/^86^Sr than their respective bulk values whereas those of samples 187 and 1977/43 are similar. The ^87^Sr/^86^Sr of the carbonate fractions of samples 187, 1977/7 and 1977/43 and their previously reported values^23^ are the same within error despite the difference in the leaching procedure used, and are also similar to the recent estimate of Archaean seawater ^87^Sr/^86^Sr ref.^29^.

**Table 1:** Lead and Sr isotopic ratios of Archaean limestones with the low ^87^Sr/^86^Sr of coeval seawater.

Sample	^206^Pb/^204^Pb	^207^Pb/^204^Pb	^208^Pb/^204^Pb	^87^Sr/^86^Sr	^87^Sr/^86^Sr*
184_bulk_	24.864	16.107	39.335	0.710224	
184_carb f_	27.208	16.206	39.551	0.702060	0.70217
185_bulk_	23.670	16.061	40.012	0.710354	
185_carb f_	24.404	16.094	40.354	0.701980	0.70209
187_bulk_	16.437	15.253	34.113	0.701541	
187_carb f_	18.202	15.441	33.762	0.701510	0.70162
1977/7_bulk_	18.689	15.592	36.173	0.702607	
1977/7_carb f_	19.157	15.714	35.978	0.702220	0.70233
1977/43_bulk_	24.034	16.704	39.664	0.701920	
1977/43_carb f_	26.002	17.098	39.804	0.701760	0.70187

*Carbonate fraction data from refs^22,23^, normalized to NBS987 ^87^Sr/^86^Sr = 0.710254. Analytical errors are ±0.000018 for ^87^Sr/^86^Sr, ±0.002 for ^206^Pb/^204^Pb, ±0.003 for ^207^Pb/^204^Pb and ±0.010 for ^208^Pb/^204^Pb; 2σ precisions for individual Sr and Pb measurements are better than these.

The ^206^Pb/^204^Pb and ^207^Pb/^204^Pb of bulk and carbonate fractions of our samples plot with or at the extension of the wide ^206^Pb/^204^P-^207^Pb/^204^Pb array of the carbonate fractions of Manjeri limestones^24^ that also occur in the Zimbabwe Craton and of Nsuze limestones^26^ and Transvaal dolomites^30^ in the Kaapvaal Craton (Fig. 1A). Notably, oceanic basalts plot within this array. The array, in turn, plots below the arrays of the carbonate fractions of Cheshire limestones^25^ as well as of surface (plotted) and very radiogenic drill core (not shown) samples from Mushandike stromatolites^24^, all in the Zimbabwe Craton. In detail, the ^206^Pb/^204^Pb and ^207^Pb/^204^Pb of three Mosher limestones (184, 185, 1977/43), together with some of the Transvaal dolomites, are more radiogenic than OIB. Finally, the ^208^Pb/^204^Pb for given ^206^Pb/^204^Pb of bulk limestones, their carbonate fractions and those of many Archaean limestones are highly variable, but for many samples they are lower than those of oceanic basalts (Fig. 1B). Significantly, some Cretaceous serpentinites and 1.9 Ga altered oceanic crust have unusually low ^208^Pb/^204^Pb, clearly indicating the extremely low Th content and, hence, Th/U of oxygenated seawater^18,31^.

The systematically high ^206^Pb/^204^Pb plus ^207^Pb/^204^Pb and low ^208^Pb/^204^Pb of the carbonate fractions we analyzed clearly indicate that some Archaean limestones have long time-integrated high μ and low κ refs^18,21^. A possibility is that such limestones precipitated locally in Archaean ‘oxygen oases’ in shallow-platforms^27,32^. Indeed, the very radiogenic Pb isotopes of the aforementioned Archaean Mushandike limestones were most probably generated in a shallow restricted basin with variable, but limited communication with the open ocean^24^. Alternatively, they may have been formed during localized concentrations or ‘whiffs’ of oxygen in the Archaean^33^, as proposed for the Nsuze metasediments^34^. Whichever the case, marine carbonates that precipitated in equilibrium with oxygenated seawater theoretically can have extremely high μ and low κ ref.^35^. Accordingly, the radiogenic Pb isotopes of some Archaean carbonates and interlayered metasediments are most probably due to in-growth of uranogenic ^206^Pb and ^207^Pb in their carbonate fraction, rather than from contamination by a high μ Archaean crust^24–26,36^.

## Discussion

Although there is consensus that the HIMU, EM1 and EM2 end-components are recycled surface material, there is little agreement on the origin of individual end-components. A currently popular idea is that HIMU is from altered MORB whereas EM1 and EM2 are from pelagic and terrigenous sediments, respectively^2,3^. Irrespective of sources, however, the end-components almost always mix with a ‘common’ or focus zone (FOZO) component^2,3,37^. The marine carbonate recycling hypothesis not only offers an alternative origin for the HIMU signature, but also explanations for some common threads in the geochemistry of oceanic basalts, particularly their unusually high ^206^Pb/^204^Pb and ^207^Pb/^204^Pb (Fig. 1A) and the almost ubiquitous link between OIB end-components and the HIMU-like FOZO^2,3,18,37^. Our new analyses for a few select Archaean limestones lend some credence to the hypothesis.

Sample 1977/43 and its carbonate fraction as well as those of samples 184 and 185 plot at the extension of HIMU OIB in the ^206^Pb/^204^Pb and ^87^Sr/^86^Sr diagram (Fig. 2). Although such limestones appear scarce, it is noteworthy that the measured Pb and Sr isotopes of many Archaean limestone outcrops may be secondary, as these are mainly effects of later metamorphism and/or alteration^22–26^. That our Archaean carbonate fractions still show their original μ, κ and Rb/Sr features is most likely due to a combination of favorable preservation and the sluggishness of element diffusion in solid rocks^38,39^. Significantly, if a subducted Archaean oceanic slab containing carbonates and/or carbonated metasediments would partially melt in the deep mantle, carbonate-rich melt would most likely form first since calcareous metasediment has the lowest melting temperature of all lithologies in the subducting lithosphere^40,41^. Moreover, high modes of melting would occur once melting initiates, as limestone is mainly comprised of a single calcite phase^42^. The carbonate-rich melt could then infiltrate and/or metasomatize the lithospheric mantle portion of the slab, as carbonate has higher diffusion rate than basaltic melt^43,44^. Subsequent melting of carbonate-metasomatized ancient upper mantle could produce OIB melts^18,21,41^.

**Figure 3:**
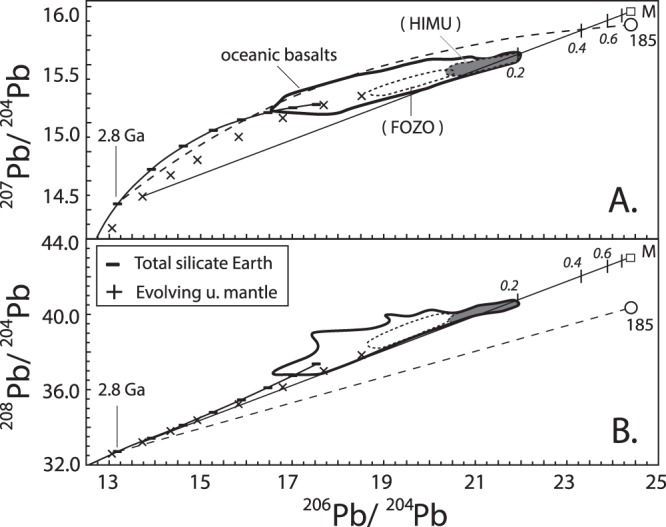
^206^Pb/^204^Pb versus A. ^207^Pb/^204^Pb and B. ^208^Pb/^204^Pb diagrams showing the evolution of the carbonate fraction of sample 185 from the 2.8 Ga Bulk silicate Earth^7^ to present (dash lines), and our model for the origin of HIMU OIB (solid lines). The model was constructed by metasomatizing (mixing) 99% of a slightly younger, 2.4 Ga evolving upper mantle (^206^Pb/^204^Pb = 13.728, ^207^Pb/^204^Pb = 14.614, ^208^Pb/^204^Pb = 33.210, 0.051 ppm Pb) ref.^7^ with 1% of a hypothetical Archaean limestone (^206^Pb/^204^Pb = 24.404, ^207^Pb/^204^Pb = 16.200, ^208^Pb/^204^Pb = 43.00; open square – M) containing the Pb of the most enriched, 8% partial melt from carbonated pelite (67.2 ppm; see ref.^41^ for details of experimental data, and modeling). The latter Pb concentration was used because of the dearth of experimental data for partial melting of limestone at high pressures. Each upper mantle and total silicate Earth symbol represents 0.4 Ga interval whereas each tick mark along the model mixing line represents 0.2 increment of melt contribution. See text for additional discussion.

To illustrate the origin of the ^206^Pb/^204^Pb and ^207^Pb/^204^Pb of HIMU OIB, we first calculated the evolution of the carbonate fraction of sample 185 (Fig. 3A). Sample 185 formed 2.8 Ga in the prevailing environment with a μ of 7.99 refs^7^ but its carbonate fraction evolved with a μ of 20.63 (dash line). We assumed the sample was part of a subducted slab that was isolated for quite some time (ca. ~b.y.) deep in the mantle to allow uranogenic ^206^Pb and ^207^Pb to grow. Then the carbonate partially melted to form a carbonatite melt that metasomatized the mantle portion of the subducted slab, which partially melted quite recently to produce plume magmas. Using these results and assumptions, we then constructed a model using a hypothetical limestone that evolved like sample 185 and relevant experimental data^41^. Metasomatism (solid line) of a subducted, evolving upper mantle^7^ (99%) by the partial melt from the limestone (1%) produced HIMU OIB containing a mere ≤20% of such melt. Notably, the metasomatic melt was highly enriched in ^206^Pb and ^207^Pb as it came from a limestone that only had U but no Pb. Moreover, the subducted slab was very low in Pb to begin with as its Pb was sequestered during subduction^2,18^ and/or later by mantle sulfides^8^, making the slab susceptible to contamination or spiking by the uranogenic Pb refs^18,21^. On the other hand, the low ^87^Sr/^86^Sr of HIMU OIB is simply that of the Archaean limestone, which had the ^87^Sr/^86^Sr of coeval seawater that, in turn, was being buffered by the upper mantle^22,23^ (Fig. 2).

Our model illustrates a similar, idealized origin for the ^208^Pb/^204^Pb of HIMU OIB (Fig. 3B), but this is an over-simplification. Sample 185 formed in the prevailing environment with a κ value of 3.99 ref.^7^ but evolved with a lower value of 2.49 (dash line), similar to many Archaean limestones (Fig. 1B) and/or serpentinite and altered crust^31^ that have extremely low κ values. However, if our hypothetical Archaean limestone indeed fully-equilibrated with oxygenated seawater, then the carbonatitic melt contribution to the ^208^Pb/^204^Pb of HIMU OIB is negligible because it had none to very little thorogenic ^208^Pb and, compared to Pb, Th was sequestered much less from the slab during subduction. Thus, the ^206^Pb/^204^Pb-^208^Pb/^204^Pb evolution of HIMU OIB (solid line) is primarily an extension of the upper mantle^7^. This notion is consistent with geochemical data indicating a uniform Th/U ratio for the ancient upper mantle^7^ and modern DMM refs^21,45,46^.

Our model also implies that mixing between an even smaller amount of Archaean (or perhaps younger) limestone and ancient upper mantle can produce the variable and radiogenic Pb and Sr isotopic ratios of the FOZO component^18,21^. On the other hand, the HIMU-like ^206^Pb/^204^Pb and radiogenic ^87^Sr/^86^Sr of the bulk of OIB can be produced through mixing between ancient mantle and carbonated crust plus sediment (e.g., containing ankerite^23^). In this case, mixing between ancient mantle (FOZO) and carbonated, variably altered MORB plus mafic (EM1) ref.^47^ to silicic (EM2) ref.^48^ metasediment are most likely involved (see also ref.^41^). Finally, mixing between carbonatitic melts from the present-day down-going slab that intersects the solidus for carbonated MORB at ca. 300 to 700 km ref.^49^ and DMM can produce the radiogenic Pb and Sr isotopes of modern enriched-MORB. Thus, recycling of marine carbonates can also solve the 1^st^ Pb paradox^21^.

We additionally propose that the Th deficiency in many ancient carbonates produced the limited variation of the κ values of OIB that hover around the BSE κ, as these are primarily extensions of the evolving upper mantle (Fig. 3B). The homogeneous DMM κ value deduced from MORB ^206^Pb/^204^Pb and ^208^Pb/^204^Pb is also an upper mantle signature^2,3,14,16^. However, the fairly recently decoupled low Th/U of MORB (i.e., 2^nd^ paradox)^13^ comes from the aforementioned carbonatitic melts from the down-going slab^18,49^ that are mainly from Proterozoic to Phanerozoic limestone, as melts from carbonated sediment has high Th/U ref.^41^. The occurrence of carbonatitic melts containing U in DMM is consistent with the isotopically distinct, high ^238^U/^235^U of MORB ref.^17^. The ‘exotic’ ^238^U/^235^U is formed in the oceanic crust at the bottom of the modern oxic ocean and subducted into the mantle, but must have been recycled back relatively quickly to the surface^17^.

In summary, our results indicate the existence of Archaean carbonates with the appropriate Pb and Sr isotopes to generate the HIMU source, per the marine carbonate recycling hypothesis. Through qualitative modeling, we illustrate via mixing between recycled upper mantle and partial melts from 1) select ancient marine carbonates the compositional signature of HIMU OIB, and 2) ancient carbonated crust and sediment the solutions to both 1^st^ and 2^nd^ Pb paradoxes. Notably, a recent study also concludes that the high U/Pb and U/Th content of ancient seawater as the source of the Pb isotopic signature of the HIMU end-component^31^. Instead of Archaean carbonatitic melt, however, the study proposes metasomatism of the source with U-enriched, supercritical liquid derived from Proterozoic (1.9 Ga) seawater. Incidentally, Proterozoic seawater would also impart a higher ^87^Sr/^86^Sr (>0.704)^22,23^ to the HIMU source. The study also locates the HIMU source domains roughly in the mid-mantle, where they can be fortuitously entrained by upwelling mantle plumes^31^. We acknowledge that the hypothesis is inconsistent with some existing data and prevailing interpretations. Foremost among these are that carbonates should break and dissolve completely during subduction^50,51^, there are very few Archaean carbonate outcrops that posses the putative HIMU composition, and carbonate melts^41^ and carbonatites^52,53^ are generally depleted in Zr, Hf and Ti but these are not possessed by HIMU OIB ref.^2^. However, there are also data indicating the occurrence of carbonate-sourced diamonds deep in the mantle^54,55^ and that carbonates can be preserved to great mantle depths^56^. Moreover, HIMU OIB is only a small fraction of OIB ref.^3^. Caution must also be taken when equating the current outcrops as true representations of the actual amount of subducted Archaean carbonates. Finally, our model indicates that the HIMU plume source contains only a minute amount of carbonatitic melt, and higher degrees of partial melting of a silicate-dominated source should increase the Zr, Hf and Ti contents of the resultant silicate melt^41,57^. Thus, more data are needed to test our hypothesis.

## Methods

The limestone samples were broken into cm-sized fragments, washed with distilled water, dried, and pulverized in an agate mortar and pestle. About 50 mg of the limestone powders were completely dissolved using a mixed, ultra-pure HF:HNO_3_ solution in Teflon beakers and then analyzed for bulk limestone Pb and Sr isotopic composition following the established sample purification and thermal ionization mass spectrometry procedure at the Scripps Institution of Oceanography^58^. To analyze the Pb and Sr isotopic composition of carbonate fractions, ca. 100 mg of powders were leached with 3 ml 1 M ultra-pure acetic acid in Teflon beakers, ultrasonicated for 15 minutes and left to react at room temperature for ca. 24 h. Then the mixtures were centrifuged and supernates were carefully separated and dried down. Afterwards, ca. 0.5 ml ultra-pure, concentrated HNO_3_ was added to the supernates to oxidize any organics. The samples were dried down again prior to Pb and Sr purification and isotopic analysis following the same above procedure. Note that the separation procedure for carbonate fraction used in this study differs from that employed in refs^22,23^ in the use of mild acetic acid rather than hydrochloric acid.

Strontium and Pb isotopic ratios were analyzed using a 9-collector, Micromass Sector 54 thermal ionization mass spectrometer. Total procedural blanks are 35 pg for Sr and 60 pg for Pb. Strontium isotopic ratios were fractionation-corrected to ^86^Sr/^88^Sr = 0.1194 and are reported relative to ^87^Sr/^86^Sr = 0.710254 + 0.000018 (n = 22) for NBS 987. Lead isotopic ratios were analyzed using the double-spike method to correct for mass fractionation during analysis; separate measurements of spiked and unspiked samples were made on different aliquots from the same dissolution. The SBL-74 ^207^Pb–^204^Pb double-spike from the University of Southampton was used, producing the following results for NBS981: ^206^Pb/^204^Pb = 19.9282 ± 0.0023, ^207^Pb/^204^Pb = 15.4870 ± 0.0031 and ^208^Pb/^204^Pb = 36.6952 ± 0.0097 (n = 7). These values and those of the samples were adjusted to ^206^Pb/^204^Pb = 19.9356, ^207^Pb/^204^Pb = 15.4891 ± 0.0031 and ^208^Pb/^204^Pb = 37.7006 ref.^59^. 2σ precisions for individual Sr and Pb measurements are better than the analytical errors for NBS987 and NBS981.

## Data Availability

All data generated or analyzed during this study are included in this published article.
